# Unveiling the vital role of soil microorganisms in selenium cycling: a review

**DOI:** 10.3389/fmicb.2024.1448539

**Published:** 2024-09-11

**Authors:** Zhihui Jiang, Zhiyong Wang, Yong Zhao, Mu Peng

**Affiliations:** ^1^Hubei Key Laboratory of Biological Resources Protection and Utilization, Hubei Minzu University, Enshi, China; ^2^College of Biological and Food Engineering, Hubei Minzu University, Enshi, China; ^3^College of Life Science, Baicheng Normal University, Baicheng, China

**Keywords:** selenium, soil, microorganisms, biogeochemical cycling, selenium reduction

## Abstract

Selenium (Se) is a vital trace element integral to numerous biological processes in both plants and animals, with significant impacts on soil health and ecosystem stability. This review explores how soil microorganisms facilitate Se transformations through reduction, oxidation, methylation, and demethylation processes, thereby influencing the bioavailability and ecological functions of Se. The microbial reduction of Se compounds, particularly the conversion of selenate and selenite to elemental Se nanoparticles (SeNPs), enhances Se assimilation by plants and impacts soil productivity. Key microbial taxa, including bacteria such as *Pseudomonas* and *Bacillu*s, exhibit diverse mechanisms for Se reduction and play a substantial role in the global Se cycle. Understanding these microbial processes is essential for advancing soil management practices and improving ecosystem health. This review underscores the intricate interactions between Se and soil microorganisms, emphasizing their significance in maintaining ecological balance and promoting sustainable agricultural practices.

## 1 Introduction

Se is a vital trace element with significant roles in both geochemistry and biological systems (Sharma et al., 2015; Wang et al., 2022e). In soil ecosystems, microorganisms are crucial in regulating the availability and cycling of Se, which in turn influences its geochemical behavior and bioavailability (Wells and Stolz, 2020). This trace element is an essential component of critical enzymes involved in oxidation–reduction reactions that maintain cellular homeostasis in various organisms (Wells et al., 2021). These processes contribute to antioxidant defenses and reduce oxidative stress (Ekumah et al., 2021). By participating in nutrient cycling, Se not only impacts the metabolic activities of soil microorganisms and plant growth but also contributes to biodiversity and ecosystem stability by modulating the survival and reproduction of various organisms across trophic levels (Graves et al., 2019; Wang et al., 2022e). In addition to its ecological importance, Se is crucial for human health, supporting antioxidative, immunomodulatory, and anticarcinogenic functions, as well as playing a role in detoxification processes (Mehri, 2020; Zhao et al., 2023). Deficiencies in Se can lead to a range of health issues, including compromised immune function, thyroid dysfunction, and cardiovascular diseases (Rahman et al., 2019; Crespo et al., 2024). Therefore, the diverse roles of Se have profound implications for both ecosystem function and human health.

In soil, Se mainly exists as selenate, selenite, or organic Se compounds (methylated compounds, selenoamino acids, selenoproteins, and their derivatives) (Pyrzyńska, 1996; Ullah et al., 2019). Microorganisms regulate the form and availability of Se through reduction and oxidation processes (Srivastava et al., 2023), which are essential for maintaining soil health and productivity (Gómez-Gómez et al., 2019). For instance, some microorganisms reduce Se from higher oxidation states to more accessible forms for plants, while others oxidize organic Se into inorganic forms, facilitating its cycling within the ecosystem (Fischer et al., 2020).

Microbially mediated Se cycling not only affects soil content and plant uptake but also has broader implications for ecosystem services and food chain dynamics (Khanna et al., 2023). As Se accumulates in food chains, microbial processes such as reduction, methylation, and demethylation regulate its conversion into bioavailable or less toxic forms. These processes influence the movement of Se between soil, plants, and animals, ultimately affecting the ecological balance, species survival, and overall health of entire ecosystems (Wang et al., 2022e). Given the significant role of microorganisms in Se cycling and ecosystem functioning, we provide a brief overview of the current state of knowledge on microbial processes involved in Se transformations and their ecological implications.

## 2 Microbial transformation and utilization of Se

**Figure 1:**
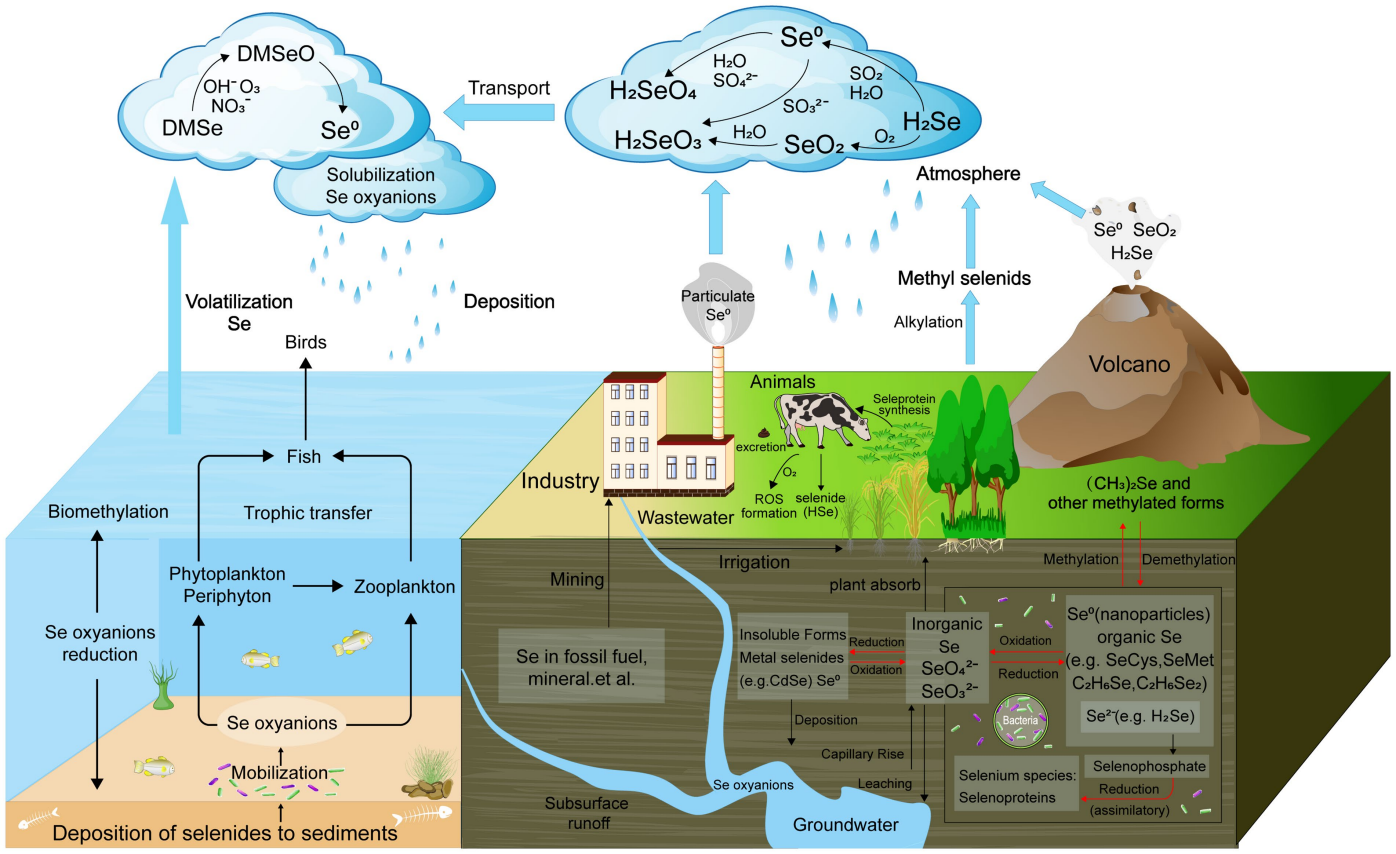
Schematic biogeochemical behavior of Se in the ecosystem. The four known biological transformations of Se—reduction, oxidation, methylation, and demethylation are marked with red.

In soil, Se predominantly occurs in two soluble inorganic forms: selenate (SeO_4_^2−^, Se^6+^) and selenite (SeO_3_^2−^, Se^4+^) (Sharma and Sharma, 2021). Additionally, Se is present as part of organic compounds, including selenocysteine, selenomethionine, and selenocysteine, which are important for its biological cycling (Petrović, 2021). Microorganisms play a vital role in transforming inorganic Se compounds into insoluble elemental Se (Se^0^), which can be filtered out of drainage water (Zhou et al., 2020). Additionally, they reduce Se compounds such as selenate, converting them into more easily absorbed organic forms such as methyl selenocysteine (Liao et al., 2024). These organic forms are readily absorbed by plants, thereby entering the food chain. Microbial communities exhibit variability in Se bioavailability, using various mechanisms and metabolic pathways to detect and utilize Se. The four known biological transformations of Se—reduction (both assimilatory and dissimilatory), oxidation, methylation, and demethylation—play a crucial role in Se cycling within ecosystems (Figure 1) (Khanna et al., 2023; Chilala et al., 2024).

### 2.1 Se-reducing bacteria

**Figure 2:**
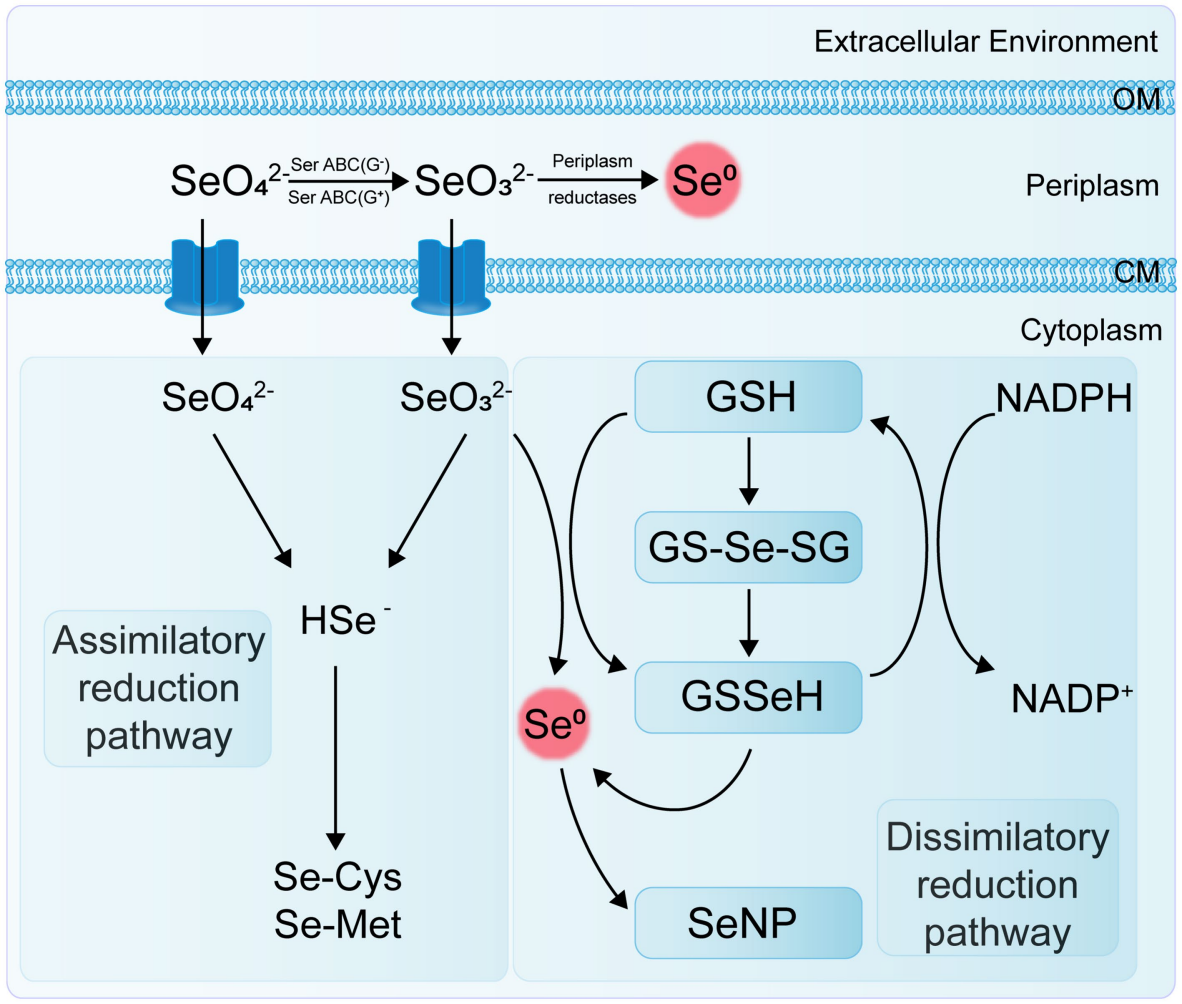
Flowchart of assimilatory and dissimilatory reduction pathways of Se.

Se-reducing bacteria are microorganisms capable of reducing inorganic Se compounds from higher to lower oxidation states. These Se reduction processes can be categorized into two main types: assimilatory reduction and dissimilatory reduction (Figure 2). Assimilatory reduction involves converting Se(VI) or Se(IV) into Se-containing amino acids and selenoproteins. In contrast, dissimilatory reduction converts Se(VI) and Se(IV) into SeNPs or selenides. Both processes promote Se cycling and enhance its bioavailability in soil ecosystems.

#### 2.1.1 Microbial assimilatory reduction of Se

The end products of the microbial assimilatory reduction of Se are Se-containing amino acids and selenoproteins, produced through specific and non-specific pathways. The primary Se-containing amino acids are selenocysteine (Sec or SeCys, U, the 21st amino acid) and selenomethionine (SeMet, the 22nd amino acid). The biosynthesis of SeCys is specific and specialized (Kieliszek et al., 2020), while SeMet can non-specifically replace methionine in protein synthesis. Se is an essential element for various organisms across almost all bacterial phyla. Bacteria capable of Se assimilation are widely distributed, with at least one-third of over 5,200 sequenced bacterial species possessing Se assimilation capabilities (Zhang et al., 2022b). Two key enzymes identified in the Se assimilation process in eukaryotes, bacteria, and archaea are selenocysteine synthase (SelA) and selenophosphate synthetase (SelD) (Hoffman et al., 2023). The latest research found that bacterial SelA, a homo-decameric enzyme composed of a pentamer of dimers, has a unique and highly symmetrical arrangement not seen in *Archaea* and *Eukarya* (Serrão et al., 2024). SelD interacts directly with the complex formed by SelA and SelC to supply the necessary selenophosphate for synthesizing Sec-tRNA^Sec^ (Scortecci et al., 2020). Additionally, selenocysteine-specific transfer RNA (tRNA^Sec^) and the selenocysteine insertion sequence (SECIS) are important factors involved in microbial Se assimilation (Wells et al., 2021; Manta et al., 2022).

#### 2.1.2 Microbial dissimilatory reduction of Se

The dissimilatory reduction of Se(VI) and Se(IV) by bacteria occurs widely in soil, sediment, and aquatic environments, typically producing SeNPs under both anaerobic and aerobic conditions. Various bacteria, including *Escherichia coli*, *Rhodospirillum rubrum*, *Rhodobacter sphaeroides*, *Bacillus subtilis*, *Pseudomonas fluorescens*, and *Bacillus licheniformis*, have been identified as capable of reducing selenate or selenite to SeNPs (Table 1). Ojeda et al. (2020) summarized several Se(IV) reduction pathways, including thiol group-mediated reduction reactions, the thioredoxin-thioredoxin reductase system, siderophore-mediated reduction reactions, sulfide-mediated reduction reactions, and dissimilatory reduction. Bacteria and fungi with thiol groups utilize these groups to facilitate Se reduction, while some microorganisms reduce selenate to Se compounds, thereby participating in the Se cycle.

**Table 1:** Se reduction microorganisms.

Organism	Se substrate	Se product	Reference(s)
**Bacteria**			
*Pseudomonas stutzeri* NT-1	SeO_4_^2−^,SeO_3_^2−^	Se^0^	Tendenedzai et al. (2021)
*Pseudomonas* sp. strain CA-5	SeO_3_^2−^	Se^0^	Yuan et al. (2024)
*Pseudomonas stutzeri* pn1	SeO_4_^2−^	SeO_3_^2−^	Staicu and Barton (2021)
*Pyrobaculum* sp. WIJ3	SeO_4_^2−^	Se^0^	Mohammadi (2021)
*Pyrobaculum arsenaticum* PZ6	SeO_4_^2−^	Se^0^	Mohammadi (2021)
*Enterobacter cloacae* SLD1a-1	SeO_4_^2−^	SeO_3_^2−^, Se^0^	Schilling et al. (2020)
*Bacillus arseniciselenatis* E1H	SeO_4_^2−^	SeO_3_^2−^	Al-Hagar et al. (2021)
*Bacillus selenitireducens* MLS10	SeO_3_^2−^	Se^0^	Al-Hagar et al. (2021)
*Bacillus beveridgei* strain MLTeJB	SeO_3_^2−^	Se^0^	Al-Hagar et al. (2021)
*Ralstonia metallidurans* CH34	SeO_3_^2−^	Se^0^	Roux et al. (2001)
*Chrysiogenetes* S5	SeO_4_^2−^	Se^0^	Staicu and Barton (2021)
*Deferribacteres* S7	SeO_4_^2−^	Se^0^	Staicu and Barton (2021)
*Pelobacter seleniigenes* KM^7^	SeO_4_^2−^	Se^0^	Staicu and Barton (2021)
*Thauera selenatis*	SeO_4_^2−^, SeO_3_^2−^	SeO_3_^2−^,Se^0^	Staicu et al. (2024)
*Thauera selenatis* AXT	SeO_4_^2−^, SeO_3_^2−^	Se^0^	Lampis and Vallini (2021)
*Sulfurospirillum barnesii* SES-3	SeO_4_^2−^, SeO_3_^2−^	Se^0^	Afkar 2012
*Bacillus cereus* CM100B	SeO_3_^2−^	Se^0^	Ashengroph and Hosseini (2021)
*Bacillus megaterium* BSB6 and BSB12	SeO_3_^2−^	Se^0^	Nagar et al. (2022)
*Bordetella petrii*	SeO_4_^2−^	Se^0^	Zhang et al. (2022a)
*Selenihalanaerobacter shriftii* DSSe-1	SeO_4_^2−^	Se^0^	Lampis and Vallini (2021)
*Rhodospirillum rubrum*	SeO_3_^2−^	Se^0^	Kessi et al. (2022)
*Rhodobacter capsulatus* B10	SeO_3_^2−^	Se^0^	Kessi et al. (2022)
*Rhodobacter sphaeroides*	SeO_3_^2−^	Se^0^	Adnan et al. (2021)
*Rhodopseudomonas palustris* N	SeO_4_^2−^, SeO_3_^2−^	Se^0^	Surachat et al. (2022)
*Clostridium* sp. BXM	SeO_4_^2−^, SeO_3_^2−^	Se^0^	An and Kim (2022)
*Shewanella oneidensis* MR-1	SeO_3_^2−^	Se^0^	Zhang et al. (2023b)
*Shewanella* sp. HN-41	SeO_3_^2−^	Se^0^	Ho et al. (2021)
*Azospira oryzae*	SeO_4_^2−^,SeO_3_^2−^	Se^0^	Zhang et al. (2020)
*Azospirillum brasilense*	SeO_3_^2−^	Se^0^	Tugarova et al. (2020)
*Veillonella atypica*	SeO_3_^2−^	Se^0^,Se^2−^	Nancharaiah and Lens (2015)
*Geobacter sulfurreducens* PCA	SeO_3_^2−^	Se^0^	Yan et al. (2023)
*Desulfovibrio desulfuricans*	SeO_4_^2−^, SeO_3_^2−^	Se^0^	Lampis and Vallini (2021)
*Desulfurispirillum indicum* sp. S5	SeO_4_^2−^, SeO_3_^2−^	Se^0^	Ojeda et al. (2020)
*Wolinella succinogenes*	SeO_4_^2−^, SeO_3_^2−^	Se^0^	Wells et al. (2021)
*Salmonella enterica serovar* Heidelberg	SeO_3_^2−^	Se^0^	Kang et al. (2022)
*Duganella* sp. strains C1 and C4	SeO_3_^2−^	Se^0^	Lampis and Vallini (2021)
*Escherichia coli*	SeO_4_^2−^, SeO_3_^2−^	Se^0^	Tendenedzai et al. (2022)
*Sedimenticola selenatireducens* AK4OH1	SeO_4_^2−^	SeO_3_^2−^,Se^0^	Lampis and Vallini (2021)
**Archaea**			
*Pyrobaculum ferrireducens*	SeO_4_^2−^, SeO_3_^2−^	Se^0^	Lampis and Vallini (2021)
*Halorubrum xinjiangense*	SeO_3_^2−^	Se^0^	Nagar et al. (2022)

Transposon mutagenesis genetic analysis has revealed that the selenate reductase in *E. coli* is a molybdoenzyme and that the reduction of selenite to Se^0^ and the reduction of selenate to selenite are two independent processes (Bébien et al., 2002). Kuroda et al. (2011) also found that the strain *B. selenatarsenatis* SF-1 independently reduces selenate to selenite through anaerobic respiration. This process is facilitated by a selenate reductase complex, with the operon SrdBCA displaying typical features of a membrane-bound and molybdopterin-containing oxidoreductase capable of encoding a respiratory selenate reductase complex. To date, a specific selenate reductase has only been purified from the Gram-negative β-proteobacterium *Thauera selenatis*, which uses selenate as the terminal electron acceptor for anaerobic respiration (Mohapatra et al., 2022). However, the biological mechanisms and enzymes involved in other selenate and selenite reduction processes remain to be characterized.

The genus *Clostridium* includes strains of anaerobic bacteria capable of reducing Se. These bacteria typically inhabit anoxic or microaerophilic environments, utilizing thiol groups or other reducing agents to convert selenate to selenides (Figueiredo et al., 2020). The genus *Desulfovibrio* comprises sulfate-reducing bacteria (SRB) that usually thrive in environments rich in organic matter and sulfur. These bacteria also possess the capability to reduce Se, using reducing agents such as hydrogen sulfide to transform selenate into selenides (Gao et al., 2023). Both archaeal and bacterial domains can use selenate and selenite as terminal electron acceptors. Under anaerobic conditions, they reduce soluble selenate and selenite to insoluble elemental Se through dissimilatory reduction (Xu et al., 2023). For example, Se-reducing bacteria can use carbon sources and electron donors to reduce Se oxyanions to elemental Se (Sinharoy and Lens, 2022); *Enterobacter cloacae* SLD1a-1 reduces selenate in the periplasmic space to SeNPs using a membrane-bound Se (VI) reductase, with SeO₄^2−^ as an alternative electron acceptor, and rapidly expels the nanoparticles (Escobar-Ramírez et al., 2021).

Under aerobic or microaerobic conditions, various bacterial strains can reduce selenate and selenite to elemental Se or selenides as a detoxification mechanism. Se-tolerant aerobic bacteria of the genus *Bacillus* (EU573774.1), isolated from Se-rich rhizosphere soil, reduce Se(IV) under aerobic conditions to produce amorphous α-Se^0^ nanospheres. This demonstrates the potential of this *Bacillus* isolate for neutralizing toxic Se(IV) anions in the environment (Hashem et al., 2021). The adsorption of selenite onto *B. subtilis* cells is mediated by thiol sites on the cell envelope, resulting in the formation of reduced organic Se compounds (e.g., R1S–Se–SR2) (Yu et al., 2018). The environmental bacterial isolate *Stenotrophomonas maltophilia* actively reduces SeO₄^2−^ and SeO₃^2−^ to red, amorphous Se^0^ and produces volatile alkyl selenides such as dimethyl selenide (DMSe), dimethyl selenyl sulfide (DMSeS), and dimethyl diselenide (DMDSe) (Lashani et al., 2023). The SeNPs synthesized by the strain *S. maltophilia* SeITE02 exhibit significant antibacterial and antibiofilm activity against various pathogens, including both reference strains and clinical isolates (Piacenza et al., 2023). Members of the genus *Rhizobium* can reduce selenite to Se^0^ under both aerobic and denitrifying conditions via molybdenum-containing proteins. However, they do not reduce selenate nor use selenite or selenate as terminal electron acceptors (Nikam et al., 2022).

Strain E1H (*B. arsenicoselenatis*) is an anaerobic bacterium characterized by spore-forming rods and grows by reducing Se(VI) to Se(IV). Strain MLS10 (*B. selenitireducens*) is a microaerophilic Se-reducing bacillus that grows by reducing Se(IV) to Se^0^. When co-cultured, these strains can completely reduce Se(VI) to Se^0^ (Boltz and Rittmann, 2021). Various phototrophic bacteria in redox steady states, such as *Rhodospirillum rubrum*, can also reduce Se salts and selenite to elemental Se, which is then excreted through the membrane and cell wall (Tugarova et al., 2020). The genus *Selenihalanaerobacter* consists of anaerobic bacteria specialized in Se utilization, capable of using selenate as a terminal electron acceptor. Through selenate reduction, these bacteria convert it to selenite and elemental Se, playing a significant role in Se cycling (Gonzalez-Gil et al., 2016; Yadav et al., 2023).

Different types of Se-reducing bacteria exhibit various mechanisms for Se reduction, and even a single bacterium may use multiple mechanisms. Current research indicates that enzymes involved in Se reduction include dehydrogenase I, sulfite reductase, nitrite reductase (NiR), and dimethyl sulfoxide reductase (Khanna et al., 2023). Reddy and Bandi (2022) discovered that the Se-reducing bacterium *Pseudomonas seleniipraecipitans* can reduce selenate to Se^0^ through nitrate reductase (NR) and that glutathione reductase and thioredoxin reductase may also be involved in the reduction of Se(IV). *Selenomonas ruminantium* reduces selenite to red elemental Se by incorporating it into selenocysteine, selenohomocysteine, selenomethionine, and selenoethionine (Hendawy et al., 2021). *Ralstonia metallidurans* CH34 (formerly known as *Alcaligenes* CH34) is a soil bacterium noted for its ability to grow in the presence of various heavy metals. It has been reported that strain CH34 can resist up to 6 mM selenite and, during the lag phase, adapts to selenite stress by reducing selenite to red elemental Se (Mohammadi and Baldwin, 2024).

The non-enzymatic reduction of Se(IV) primarily involves sulfide-mediated reduction and thiol compound-mediated reduction, such as by reduced glutathione (GSH). Studies have demonstrated that the abiotic reduction of selenite by glutathione produces reactive oxygen species (ROS). This indicates that glutathione is involved in the reduction of selenite to elemental Se, as mediated by *Rhodospirillum rubrum* and *E. coli* (Wang et al., 2022a). Additionally, Se reduction in some bacteria may be associated with extracellular secretions. For instance, in *Pseudomonas aeruginosa* strain JS-11, the metabolite pyocyanin-1-carboxylic acid (PCA) and known redox agents (such as NADH and NADH-dependent reductases) are responsible for reducing SeO₃^2−^ to Se^0^ (Xue et al., 2024). These Se-reducing bacteria exhibit diverse ecological characteristics and physiological properties, and their distribution and activity in the soil significantly impact Se cycling and bioavailability. Research on these bacteria enhances our understanding of the biogeochemical cycle of Se and the functioning of soil ecosystems.

### 2.2 Se-oxidizing Bacteria

Se-oxidizing bacteria are microorganisms capable of oxidizing organic Se compounds or inorganic Se compounds from lower to higher oxidation states. These bacteria include heterotrophs, sulfur-oxidizing bacteria, and possibly fungi (Luo et al., 2022). They play a crucial role in soil and water environments, influencing the bioavailability and cycling of Se. Sarathchandra and Watkinson (1981) discovered that *Bacillus megaterium*, isolated from soil, could oxidize elemental Se in laboratory cultures to selenite and trace amounts of selenate (with selenite content <1%). This observation represents an important but previously unreported oxidation step in the biological Se cycle. To date, few studies have demonstrated that the oxidation of Se^0^ in soil is predominantly biological and occurs at a relatively slow rate. There is limited research on the types of Se-oxidizing bacteria, particularly pure cultures capable of oxidizing Se^0^, and the biosynthetic mechanisms of Se oxidation (Wasserman et al., 2021). This may be because microbial oxidation in the soil is partly limited by the adsorption of Se(IV) on the soil surface, and the process of Se^0^ being oxidized by microorganisms to higher oxidation states of Se is very slow (Wang et al., 2022a).

Additionally, studying the oxidation of Se^0^ by soil microorganisms is crucial for increasing the amount of bioavailable Se in soil, enhancing Se enrichment in plants, and ultimately supplementing Se in the human diet. *Agrobacterium* sp. strain T3F4 can oxidize Se^0^ to Se(IV), establish stable colonies in the soil, and dissolve soil-bound Se(IV), thereby enhancing Se bioavailability and promoting Se uptake in crops (Zhu et al., 2021). Guo et al. (2024a) demonstrated that *Agrobacterium* T3F4 enhances the absorption of Se by cabbage plants by oxidizing Se in the soil, thereby increasing its bioavailability. Four Se-oxidizing bacteria, namely *Dyella* spp. LX-1 and LX-66, and *Rhodanobacter* spp. LX-99 and LX-100, isolated from seleniferous soil, were involved in the oxidation of SeMet, SeCys2, selenourea, and Se^0^ to Se(IV) in pure cultures (Luo et al., 2022).

Some strains of the genus *Thiobacillus* can oxidize Se independently. They typically inhabit sulfur-rich environments and use sulfur oxidase to oxidize Se^0^ to selenate or other higher oxidation state Se compounds. *Thiobacillus* ASN-1 primarily produces Se(VI) rather than Se(IV) as the oxidation product of Se^0^. Intermediate metabolites from Se^0^ oxidation, such as volatile fatty acids, hydrogen, and methane, are utilized by heterotrophic Se(VI) reducers for Se(VI) detoxification (Li et al., 2022). Kushwaha et al. (2022) found that acetate, glucose, and sulfide promoted Se oxidation, confirming the involvement of chemoautotrophic and chemoheterotrophic species of *Thiobacillus*. *Leptothrix* sp. MnB-1 can oxidize Se^0^ to Se(VI) through complex mechanisms, though its enzymes and pathways have not yet been reported (Guo et al., 2023).

### 2.3 Microbial transport of Se

Some microorganisms actively absorb and transport Se through specific membrane proteins or carriers, acquiring Se from the environment or surrounding organic matter. Current research generally agrees that Se(VI) is transported via sulfate [S(VI)] transport channels, while Se(IV) can be transported through various mechanisms. Kinetic studies by Goff (2020) on the transport of selenite, selenate, and sulfate in wild-type *E. coli* K-12 indicate that K-12 shares the same transport system for Se and sulfate. Microorganisms produce specific enzymes, known as selenoenzymes, during the absorption and transport process, converting inorganic Se or organic Se compounds into forms that can be utilized by microbial cells (Chaudière, 2023). In yeast, Kieliszek et al. (2020) reported that the proton-coupled monocarboxylate transporter Jen1p in *Saccharomyces cerevisiae* transports selenite into the cell in a proton-dependent manner, similar to the transport mechanism for lactate. This facilitates high-affinity uptake of selenite and promotes its accumulation within the cell.

Lazard (2021) discovered that in *S. cerevisiae*, the uptake of selenite is influenced by the phosphate concentration in the growth medium. Both high- and low-affinity phosphate transport proteins are involved in this process. Under low-phosphate (Pi) conditions, the high-affinity transporter Pho84p primarily mediates selenite uptake. When phosphate is abundant, selenite is absorbed through low-affinity transporters, such as Pho87p, Pho90p, and Pho91p. This shift occurs because the high-affinity transport, which includes Pho84p and Pho89p, is upregulated by the phosphate signal transduction pathway (PHO) during Pi starvation. Pho84p operates most effectively at neutral to acidic pH, while Pho89p functions at alkaline pH. In contrast, the low-affinity transporters Pho87p, Pho90p, and Pho91p, which have a lower affinity for phosphate, are constitutively expressed but their activity is post-transcriptionally downregulated in low-phosphate conditions by Spl2p, a component of the PHO regulon.

The transport mechanism of Se^0^ in bacteria remains unclear, and specific selenite transport proteins have not been identified. Studies by Peng et al. (2021) indicate that *E. coli* K-12 can transport sulfate, selenate, and selenite via ABC transporters, but this system has a binding affinity for sulfate that is five times stronger than for selenate and 37 times stronger than for selenite. Se(VI) reductase is a trimeric complex with structural similarities to other enzymes, though it also exhibits some detailed differences. For example, in *T. selenatis* AXT, the Se(VI) reductase SerABC is located in the periplasmic space, exhibits high substrate specificity, and produces SeNPs that accumulate in the cytoplasm or extracellularly (Lampis and Vallini, 2021). The SerA subunit is a 96 kDa catalytic subunit containing a molybdenum cofactor, SerB is a 40 kDa iron–sulfur protein containing one [3Fe-4S] cluster and three [4Fe-4S] clusters, and SerC is a 23 kDa protein containing a heme b cofactor (Staicu and Barton, 2021). The genes encoding these three subunits, serA, serB, serC, and serD, form an operon, with serD encoding a system-specific molecular chaperone protein involved in the insertion of the molybdenum cofactor into SerA (Wang et al., 2022b). Electrons are transferred through quinol cytochrome c oxidoreductase (QCR) or quinol dehydrogenase (QDH) to cytochrome c4 in the periplasm, and then to the cytochrome b of the Se(VI) reductase complex, specifically to the SerC subunit of SerABC (Figure 2).

It has been reported that ABC transporters play a significant role in the mechanisms of Se uptake, metabolism, and tolerance in various plants (Jiao et al., 2022). For instance, in tea plants (*Camellia sinensis*), exposure to selenate increases the expression of ABCC8 and ABCG14, while selenite exposure upregulates ABCA2 and ABCC4 (Ren et al., 2022). In ryegrass, ABC5 and ABC11 are induced under high-dose selenite treatment (Chen et al., 2022). In the Se hyperaccumulator *Stanleya pinnata*, ABCC2, ABCF5, and ABCG40 are believed to be involved in vacuolar sequestration and the distribution of excess Se(VI) to mitigate toxicity (Rao et al., 2021).

Arbuscular mycorrhizal fungi (AMF) have the ability to absorb sulfate. Specifically, the sulfate transporters GBC38160.1 and GBC25943.1, along with the sulfate permease PKY50973.1 in *Rhizophagus irregularis* (an AMF species), as well as the sulfate transporters EDR02618.1 and EDR02177.1, and sulfate permeases EDR11271.1 and EDR00466.1 in the ectomycorrhizal fungus *Laccaria bicolor*, have been shown to enhance the uptake and transport of sulfate to the host plants. Given that Se and sulfur (S) belong to the same group (VI-A) in the periodic table, Se exhibits chemical properties that are highly similar to sulfur. Se can be absorbed in the form of selenate or selenite and is transported to the host plant via sulfur assimilation pathways, including sulfur transporters. This process leads to the biosynthesis of SeCys, SeMet, and various Se-homologs of sulfur-containing metabolites (Ye et al., 2020).

Se-absorbing bacteria primarily include *Fibrobacter succinogenes*, *Ruminococcus albus*, *Streptococcus* spp., *Lactobacillus* spp., and *Prevotella ruminicola*. The transport and absorption mechanisms for Se(IV) in these bacteria differ from those for S(VI) or Se(VI) and are not influenced by the presence of S(VI) or Se(VI). In *Selenomonas*, selenite is incorporated into selenocysteine, selenoethionine, selenohomocysteine, and selenomethionine, and is also reduced to Se^0^ (Hendawy et al., 2021). The soil bacterium *Pseudomonas putida* KT2440 has been demonstrated to reduce and transport selenite under aerobic conditions through central metabolic processes, sulfur metabolism, and oxidative stress responses, leading to the production of SeNPs. Genes such as *sucA*, *D2HGDH*, and *PP_3148* indicate that the metabolism of 2-oxoglutarate and glutamate plays a crucial role in the conversion of selenite to elemental Se. Conversely, mutations affecting sulfite reductase activity significantly impair the bacteria’s ability to reduce selenite.

Additionally, genes related to sulfur metabolism (*ssuEF*, *sfnCE*, *sqrR*, *sqr*, and *pdo2*) and stress response (*gqr*, *lsfA*, *ahpCF*, and *sadI*) have been identified as key players in the selenite conversion process (Avendaño et al., 2023). In the photosynthetic bacterium *Rhodobacter sphaeroides*, selenite enters the cytoplasm mediated by a polyol transporter from red algae, but similar polyhydroxy compound transport systems have not been reported in other bacteria (Wang et al., 2022a). Additionally, Padhi and Chatterjee (2023) studied the phenotypic characteristics and selenite consumption in DedA mutants of *Ralstonia metallidurans*, indicating that DedA is involved in the transport and absorption of selenite.

### 2.4 Microbial methylation and demethylation of Se

**Figure 3:**
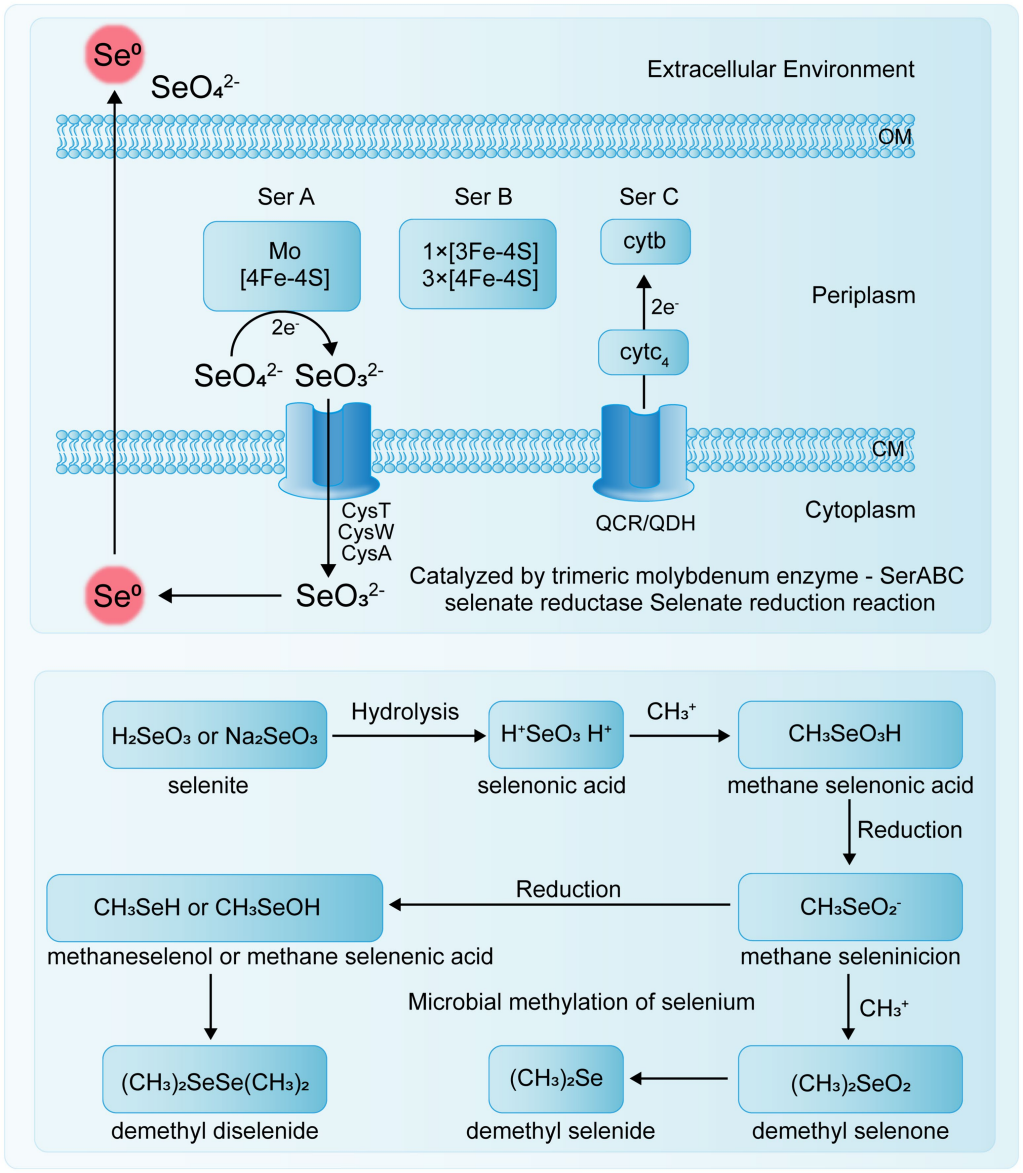
Microbial Se reduction and methylation.

Studies have shown that some microorganisms possess the ability to methylate Se, converting inorganic or organic Se compounds into methylated Se compounds, such as methyl selenate (Figure 3) (Ojeda et al., 2020). Because the resulting methylated Se compounds are often volatile, this process serves as a detoxification mechanism for soil environments and has important applications in the remediation of Se-contaminated sites. Yang et al. (2024) reviewed the mechanisms by which environmental microorganisms methylate and demethylate Se compounds, indicating that microbial methylation of reduced Se oxyanions is a potentially effective detoxification process.

For instance, *Rhodocyclus tenuis* and *Rhodospirillum rubrum* can utilize Se(VI) to form DMSe and DMDSe in photoautotrophic processes. Additionally, *R. tenuis* can produce DMSe from selenides (Pal et al., 2022). Studies have shown that if the initial Se species are Se(IV), Se(VI), or Se(0), the Se methylation process necessarily involves both reduction and methylation steps, with different microorganisms using various pathways for Se methylation (Genchi et al., 2023). In soil, inorganic Se compounds can be converted by some microorganisms into volatile methylated substances (DMSe, DMDSe, and dimethyl selenone or methyl selenite), first undergoing Se methylation followed by Se reduction (Petrović, 2021). In some bacteria, such as *Corynebacterium* sp., Se is first reduced and then methylated (Garg et al., 2020).

Several enzymes associated with Se methylation have been identified (Table 2). Thiopurine methyltransferase (TPMT) is a monomeric, cytoplasmic S-adenosylmethionine (SAM)-dependent methyltransferase that plays a crucial role in thiopurine metabolism. It catalyzes the S-methylation of aromatic and heterocyclic thiol compounds, including 6-mercaptopurine (6-MP) and thiopurine, and also facilitates the methylation of sulfur, Se, and tellurium elements (Mu et al., 2024). Bacterial thiopurine methyltransferase (bTPMT) plays a crucial role in the methylation of seleno compounds, contributing to the detoxification process by converting reactive Se species into less toxic, volatile forms such as DMSe. Ranjard et al. (2003) discovered that bTPMT and thiocoraline methyltransferase are capable of methylating selenite and (methyl) selenocysteine into DMSe and DMDSe through a homologous protein, MmtA, in *Pseudomonas* sp. Hsa.28 and *E. coli* cells. Alonso-Fernandes et al. (2023) demonstrated that the SAM-dependent methyltransferase encoded by the *TehB* gene in *E. coli* is a highly potent detoxification protein. This enzyme can detoxify oxyanions derived from the chalcogen elements tellurium and Se without differentiating between selenite and tellurite oxyanions.

**Table 2:** Se volatilizing microorganisms.

Organisms	Se substrate	Se product	Reference(s)
**Bacteria**			
*Corynebacterium* sp.	SeO_4_^2−^,SeO_3_^2−^,Se^0^	DMSe	Garg et al. (2020)
*Aeromonas* sp.	SeO_3_^2−^	DMSe, DMDSe	Afkar (2012)
*Pseudomonas fluorescens K27*	SeO_4_^2−^	DMSe, DMDSe, DMSeS	Saghafi et al. (2020)
*Rhodocyclus tenuis*	SeO_4_^2−^,SeO_3_^2−^	DMSe, DMDSe	Bosu et al. (2023)
*Aeromonas veronii*	SeO_4_^2−^,SeO_3_^2−^,SeS_2,_H_2_SeO_3_, SeH	DMSe, DMDSe, methyl selenol, DMSeS	Zayed et al. (2020)
**Fungi**			
*Scopulariopsis brevicaulis*	SeO_4_^2−^,SeO_3_^2−^	DMSe	Garg et al. (2020)
*Penicillium notatum*	SeO_4_^2−^,SeO_3_^2−^	DMSe	Zambonino et al. (2021)
*Penicillium citrinum*	SeO_3_^2−^	DMSe, DMDSe	Amin et al. (2021)
*Penicillium chrysogenum*	SeO_4_^2−^,SeO_3_^2−^	DMSe	Ruiz-Fresneda et al. (2024)
*Penicillium* sp.	SeO_3_^2−^	DMSe	Žižić et al. (2022)
*Penicillium* sp.	SeO_4_^2−^,SeO_3_^2−^	DMSe	Liang et al. (2019)
*Aspergillus niger*	SeO_4_^2−^	DMSe	Siddharthan et al. (2024)
*Schizopyllum commune*	SeO_3_^2−^	DMSe	Anusiya et al. (2021)
*Candida humicola*	SeO_4_^2−^,SeO_3_^2−^	DMSe	Zayed et al. (2020)
*Alternaria alternata*	SeO_4_^2−^,SeO_3_^2−^	DMSe	Santelli et al. (2023)
**Algae**			
*Chlorella* sp.	SeO_3_^2−^	DMSe, DMDSe, DMSeS	Hoyos et al. (2023)

To date, methanogenic bacteria, iron-reducing bacteria, and SRB are the most commonly identified microorganisms involved in demethylation processes (Du et al., 2019). Studies have shown that Se can reduce net methylation of mercury (Hg) by inhibiting SRB activity, suggesting that Se may either promote Hg demethylation or inhibit methylation by other microorganisms (Guo et al., 2024b). Microbial-mediated methylation and demethylation of Hg occur during the conversion of sulfate (SO_4_^2−^) to sulfide (S^2−^) (Wang et al., 2022c). Consequently, the transformation of SeO_4_^2−^ to Se^2−^ may similarly occur during this process, as the reactions involving Se and S share several common enzymes (Chen and Li, 2023).

### 2.5 Case studies of Se and other related microorganisms

#### 2.5.1 Symbiotic relationships enhancing Se uptake

The interactions between Se and microorganisms have garnered significant attention in the fields of environmental microbiology and ecotoxicology. Research indicates that certain mycorrhizal fungi can form symbiotic relationships with plants, facilitating the uptake and utilization of Se through symbiotic cooperation, thereby enhancing plant Se uptake from the soil (Li et al., 2020). Li et al. (2021) isolated 14 Se-tolerant endophytic bacteria from the *Cardamine hupingshanensis* in the Se-rich region of Enshi, China. These bacteria belong to 11 different genera and exhibit tolerance to various concentrations of selenite and selenate. Additionally, a study found that the mycorrhizal fungus *Glomus intraradices* can enhance Se uptake in leguminous plants, thereby increasing Se accumulation in the plants (Liu et al., 2024).

#### 2.5.2 Effects of Se on soil microbial communities

Several studies have explored the effects of Se on the structure and function of soil microbial communities. Kang et al. (2024) found that the addition of Se significantly alters the community structure of soil bacteria and fungi, leading to changes in the abundance and diversity of specific microbial strains. Rosenfeld et al. (2018) indicated that bacterial community richness and diversity decrease significantly with increased Se levels, suggesting that Se contamination shapes communities in favor of Se-tolerant microorganisms. Additional studies have focused on the impact of Se on microbial metabolism and growth. While Se is an essential trace element for microorganisms, high concentrations can be toxic, adversely affecting microbial metabolism and growth. In soils with elevated Se concentrations, Se-reducing bacteria can be utilized to convert highly toxic inorganic Se compounds into less toxic forms through bioremediation, thereby mitigating the toxic effects of Se in soil or water.

## 3 The critical role of microorganisms in Se cycling and soil remediation

### 3.1 Microbial mechanisms in Se cycling

Microorganisms are central to the biogeochemical cycling of Se in soil ecosystems, primarily through their ability to mediate redox transformations, methylation, and volatilization processes. These microbial activities convert Se between different oxidation states, such as SeO₄^2−^ and SeO₃^2−^, and into Se^0^, or volatile methylated forms. These transformations are crucial for regulating Se mobility, bioavailability, and toxicity in soils. For instance, Se-reducing bacteria can reduce selenate to elemental Se, a less toxic and insoluble form, thereby preventing the accumulation of harmful Se in the environment. Conversely, certain bacteria oxidize reduced Se forms back to SeO₄^2−^, contributing to its recycling within the ecosystem. Additionally, microorganisms such as fungi and algae can methylate Se, converting it into volatile compounds such as DMSe, which are released into the atmosphere, thus removing Se from the soil and participating in the global Se cycle (Figure 1).

### 3.2 Microbial remediation of Se-contaminated soils

With the extensive use of Se compounds in agricultural and industrial production, increasing amounts of Se (mainly SeO₄^2−^ and SeO₃^2−^) are being released into the environment, posing threats to soil safety and the natural environment. Currently, approximately 2,700 tons of Se are produced annually, but only approximately 15% is recovered (Gebreeyessus and Zewge, 2019). Therefore, there is an urgent need for efficient, cost-effective, and environmentally friendly Se remediation technologies to mitigate the environmental impact of Se and enable its recycling. Bioremediation, especially microbial remediation, offers unique advantages in cost control and recovery efficiency, making it a highly regarded approach in the study of Se pollution remediation technologies. Consequently, microbial remediation technologies are increasingly researched and applied in the treatment of Se-contaminated soils.

The accumulation and volatilization of Se by algae in surface water are important parts of the biogeochemical cycle of Se (Zhang et al., 2023a). Liu et al. (2019) demonstrated that adding six types of algae to an artificial wetland water treatment system can enhance Se removal efficiency. This method significantly reduces the risk of Se accumulation in ecotoxic forms by promoting Se volatilization. This study found that the most effective algae for Se removal was the *Chlorella vulgaris* strain (Cv). Cv primarily accumulated Se in the form of selenomethionine (a precursor to volatile Se) and Se^0^. Within 72 h, Cv removed 96% of Se (provided as selenate), with up to 61% volatilized into the atmosphere.

Selenate-respiring bacteria, such as *T. selenatis*, have been used in laboratory-scale bioreactor systems to treat refinery wastewater containing selenite. In these systems, *T. selenatis* and a significant population of denitrifying bacteria reduce selenite present in the water, along with selenite formed from the reduction of selenate. The results showed a 95% reduction in Se oxyanion content in wastewater with an initial Se concentration of 3.4 mg/L (Sinharoy and Lens, 2020).

In practical applications, *T. selenatis* has been used to treat Se-containing wastewater in a medium-scale bioreactor system in the Panoche Water District of California’s San Joaquin Valley. After reduction by *T. selenatis* in the reactor, the concentration of Se oxyanions (selenate and selenite) in the discharge water was reduced by 98%. Analysis of the reactor effluent indicated that 97.9% of the oxidized Se was converted to insoluble but recoverable Se^0^. The small Se^0^ particles produced by reduction were also recovered using a flocculant (Nalmet 8,072), with 91 to 96% of the total recovered Se being Se^0^ (Arias-Paić et al., 2022). The selenate-reducing bacterium *Bacillus* sp. SF-1, using lactate as an electron donor, can remove 41.8 mg-Se/L of selenate and excess lactate from wastewater. Strain SF-1 converts selenate to selenite and then to elemental Se through anaerobic respiration. When the cell retention time in the bioreactor was relatively short at 2.9 h, the selenate was effectively converted entirely to selenite. With a longer cell retention time, the soluble Se (selenate and selenite) was successfully reduced to non-toxic elemental Se, with a production rate of 0.45 mg/L/h of elemental Se (Baggio, 2020). Otsuka and Yamashita (2020) added *Pseudomonas stutzeri* NT-I to a 256-liter medium-scale bioreactor, utilizing its ability to reduce oxidized Se to elemental Se. This approach proved effective in detoxifying soluble Se in smelting wastewater containing Se.

### 3.3 Influence of microbial Se cycling on plant growth

Microbial transformations of Se significantly influence its availability and form, directly affecting plant uptake and health. The form of Se in soil, such as selenides or Se^0^, cannot be directly absorbed and utilized by plants (Wang et al., 2022e). Microorganisms convert these inorganic forms into more bioavailable compounds, such as selenate and selenite, which plants can readily assimilate. This microbial activity is crucial for enabling plants to benefit from Se, promoting their growth, nutritional quality, and resistance to environmental stress. Se plays multiple roles in plants, including promoting growth, enhancing resistance, and improving crop quality (Table 3).

**Table 3:** Role of Se in plant growth and physiology.

Plant	Se application results	Mechanism	Reference(s)
Wheat	Promotes plant growth	Increasing aboveground dry weight and grain yield, increasing the concentration of organic Se, and promoting plant growth.	Xia et al. (2020)
Pakchoi	Maintaining the stability of microbial communities in chromium-contaminated soil	The oxidation of chromium Cr (VI) and the reduction of Se reductase ratio increase soil pH and maintain micro stability in chromium-contaminated soil.	Cai et al. (2019)
Tomato	Enhancing resistance to soil salt stress	Regulating the antioxidant defense system in the chloroplasts of tomato seedlings to alleviate salt-induced oxidative stress and reverse the negative effects of soil salt stress.	Wu et al. (2023)
Rice	Can produce Se-rich rice	The enrichment of Se in the soil in rice endosperm through biologically enhanced grains resulted in changes in antioxidant enzyme activity and gas exchange in rice leaves.	Ramalho et al. (2020)
Corn	Relieve salt stress in corn	Exogenous Se alleviates soil salt stress in maize by enhancing photosynthetic capacity, antioxidant enzyme activity, and regulating Na^+^ homeostasis.	Abbood and Al-Shammari (2021)
Lettuce	Improve nitrogen metabolism and promote plant growth	All enzyme activities [nitrate reductase (NR), nitrite reductase (NiR), glutamine synthase (GS), and glutamate synthase (GOGAT)] have increased.	Bian et al. (2020)
Chicory	Increase the respiratory potential of seedlings	Utilize the positive role of PSII photochemistry to promote plant growth	Umami et al. (2022)

Studies have shown that the presence of Se-transforming microbes can increase the concentration of essential Se-containing compounds in plants, leading to improved antioxidant enzyme activities and enhanced tolerance to abiotic stresses. Bian et al. (2020) analyzed various enzyme activities—NR, NiR, glutamine synthetase (GS), and glutamate synthase (GOGAT)—in lettuce when different doses and forms (selenate and selenite) of Se were applied. They found that all enzyme activities increased, particularly with the application of selenite. These results indicated that appropriate concentrations of Se could promote the accumulation of starch in plant chloroplasts, benefiting plant growth. Similar conclusions were reached in studies on chicory (Umami et al., 2022) and potatoes (Li et al., 2023) when Se was applied.

Appropriate amounts of Se enhance the activities of several key enzymes, including superoxide dismutase, glutathione reductase, dehydroascorbate reductase, monodehydroascorbate reductase, glutathione peroxidase, and thioredoxin reductase. These enzymes play a crucial role in restoring photosynthetic responses and enzyme activity in the electron transport chain of stressed plants (Liu et al., 2022). The addition of suitable amounts of Se can increase the biomass and chlorophyll content of wheat seedlings as well as their antioxidant capacity. Various Se treatments have been shown to increase the content of antioxidant compounds (anthocyanins, flavonoids, and phenolic compounds) and the activities of antioxidant enzymes (peroxidase and catalase). Optimal Se supplementation can reduce the production of free radicals, decrease lipid peroxidation in membranes, promote biomass accumulation, mitigate chloroplast damage, and increase chlorophyll content (Lanza and Dos Reis, 2021). Das et al. (2022) observed that lower concentrations of Se supplementation improved the growth and arsenic resistance of crops such as rice seedings. Applying Se to rice seedings increased the activity of metallothioneins (MT), thiols, and glutathione-S-transferase (GST), thereby reducing oxidative damage caused by arsenic.

#### 3.3.1 Impact of Se cycling on soil biodiversity and ecological function

Microbial processes are crucial in driving Se cycling within soil ecosystems, significantly influencing soil biodiversity and ecological functions (Wang et al., 2022d). Through reduction, methylation, and volatilization, microorganisms convert Se into various chemical forms, impacting its bioavailability and toxicity (Figure 1). These transformations play a key role in detoxifying Se, converting harmful forms such as selenate into less toxic elemental Se or volatile compounds such as DMSe, which can be released from the soil (Moulick et al., 2024).

The presence of Se in soil can also shape microbial community structures by selecting Se-tolerant species, which may lead to shifts in biodiversity and ecosystem function. Liu et al. (2024) found that Se could successfully decrease soil Cd availability and relieve the harm of Cd in wheat by modifying rhizosphere soil microbial communities. Rosenfeld et al. (2018) suggested that Se contamination shapes communities in favor of Se-tolerant microorganisms. While these shifts can enhance the soil’s ability to cope with Se stress, they may also disrupt the balance of microbial communities, potentially affecting other ecosystem processes.

Furthermore, microbial Se cycling is interconnected with other biogeochemical cycles, such as those of sulfur, carbon, and manganese, further complicating its impact on soil health (Rosenfeld et al., 2020; Li et al., 2022). Understanding the role of microorganisms in Se cycling is vital for developing strategies to manage soil health, particularly in Se-impacted environments. Effective management of these microbial processes can enhance soil resilience, protect biodiversity, and support sustainable agricultural practices.

## 4 Future perspectives and conclusion

### 4.1 Future research directions

To effectively utilize Se-reducing or Se-oxidizing microorganisms, future research should focus on the following areas: (1) enhancing microbial resilience and efficiency: screening or engineering microbial strains with high Se reduction capabilities, tolerance to harsh environmental conditions and high Se concentrations, and ensuring ecological safety; (2) optimizing microbial remediation techniques: accelerate microbial processes and establish efficient methods for recovering Se reduction products to make remediation more time-effective; (3) understanding molecular interactions: elucidate the molecular mechanisms of microbial influence on Se redox reactions and their regulation by environmental factors; (4) exploring microbial Se interactions in diverse ecosystems: investigate microbial Se interactions across various, including extreme and human-impacted, environments to understand their ecological roles; (5) developing innovative technologies: create new strategies that leverage microbial Se interactions for improved agricultural productivity and effective remediation of contaminated soils and water; (6) expanding knowledge of Se metabolism: discover and characterize Se-oxidizing bacteria and the enzymatic processes governing Se transformations for a comprehensive understanding of Se metabolism; (7) characterizing microbial roles in Se dynamics: clarify how microorganisms affect soil Se transformations and plant Se uptake to better manage Se cycling in ecosystems; and (8) balancing reduction and oxidation processes: study both Se reduction and the less-explored oxidation processes to develop effective and balanced Se management strategies. By addressing these research directions, the goal is to develop more effective tools and strategies for managing Se in the environment, with profound implications for both environmental science and agriculture. This integrated approach will help mitigate Se-related environmental challenges while enhancing the sustainability of agricultural practices.

## 5 Conclusion

This review highlights the crucial role of soil microorganisms in the Se cycle, encompassing reduction, oxidation, methylation, and transport processes. These microbial activities are essential for regulating Se bioavailability and mitigating its toxicity, thereby maintaining soil health and ecological balance. The interactions between microorganisms and Se not only enhance soil fertility but also contribute to the resilience of ecosystems in Se-contaminated environments. Understanding these processes is vital for developing effective soil management practices and bioremediation strategies that promote sustainable agricultural and environmental outcomes. Future research should focus on elucidating the molecular mechanisms of microbial Se transformation and exploring novel microbial strains to optimize Se cycling in diverse ecosystems.
